# Analysis of the serum proteome profile of wild stump-tailed macaques (*Macaca arctoides*) seropositive for Zika virus antibodies in Thailand

**DOI:** 10.3389/fvets.2024.1463160

**Published:** 2024-11-12

**Authors:** Pakorn Ruengket, Sittiruk Roytrakul, Daraka Tongthainan, Kobporn Boonnak, Kanokwan Taruyanon, Bencharong Sangkharak, Wirasak Fungfuang

**Affiliations:** ^1^Genetic Engineering and Bioinformatics Program, Graduate School, Kasetsart University, Bangkok, Thailand; ^2^Functional Proteomics Technology Laboratory, National Center for Genetic Engineering and Biotechnology (BIOTEC), National Science and Technology Development Agency, Pathum Thani, Thailand; ^3^Faculty of Veterinary Medicine, The Rajamangala University of Technology Tawan-ok, Chonburi, Thailand; ^4^Department of Immunology, Faculty of Medicine Siriraj Hospital, Mahidol University, Bangkok, Thailand; ^5^Wildlife Conservation Division Protected Areas Regional Office 3, Department of National Parks, Wildlife and Plant Conservation, Ratchaburi, Thailand; ^6^Wildlife Conservation Division, Department of National Parks, Wildlife and Plant Conservation, Bangkok, Thailand; ^7^Department of Zoology, Faculty of Science, Kasetsart University, Bangkok, Thailand

**Keywords:** Zika virus, proteomics, stump-tailed macaque, serum, Thailand

## Abstract

Zika virus (ZIKV) is a member of the Flaviviridae virus family and poses a significant global health concern. ZIKV is transmitted by *Aedes* mosquitoes, and it has been implicated in various neurological conditions associated with fetal brain development. ZIKV has two transmission cycles: a sylvatic cycle in which nonhuman primates are infected via arboreal mosquito bites, and an interhuman (urban) cycle in which the virus is transmitted among primates by *Aedes* mosquitoes. ZIKV was first discovered in wild macaques, and the danger posed by the virus is increased due to the close proximity between humans and wild animals in modern society. However, data regarding the extent and role of infection in nonhuman primates are limited. Thus, there is an urgent need for improved surveillance, diagnostic methods, and public health interventions to effectively combat ZIKV transmission and its associated health impacts in Southeast Asia. In this study, we used a proteomics and bioinformatics approach to profile serum proteins in wild stump-tailed macaques seropositive for neutralizing antibodies against ZIKV. A total of 9,532 total proteins were identified, and 338 differentially expressed proteins were identified between naïve and seropositive animals. A total of 52 important proteins were used to construct a serum proteomic profile. These 52 important proteins were associated with immune and inflammatory responses (36.54%), neurological damage (23.08%), viral activities (21.15%), the apoptosis signaling pathway (9.61%), and other pathways (9.61%). Our proteomic profile identified proteins that inhibit the apoptosis pathway, intracellular resource competition with the virus, and neurological damage due to ZIKV and the host immune and defense responses.

## 1 Introduction

Zika virus (ZIKV) is a positive-sense, single-stranded RNA virus belonging to the Flaviviridae family (1, 2), and it is related to Dengue virus (DENV), Yellow fever virus, tick-borne encephalitis virus, and West Nile virus. ZIKV is transmitted by *Aedes aegypti* mosquitoes, but it can also be transmitted via sexual contact, in addition to perinatal and blood-borne routes (3, 4). ZIKV was first isolated in 1947 from a rhesus monkey in the Zika forest of Kampala, Uganda (5, 6). Previously, infection with ZIKV was frequently disregarded because the symptoms were mild and self-limiting, but in 2015, reports emerged that 440,000–1,300,000 people had been infected, including a patient who delivered an infant with the neurodevelopmental disorder microcephaly (7). Because ZIKV preferentially infects human neural progenitor cells, infection can result in a variety of serious neurological diseases, including meningoencephalitis, acute disseminated encephalomyelitis, myelitis, cerebrovascular complications, seizures and encephalopathy, sensory polyneuropathy, and sensory neuronopathy (8).

Southeast Asia harbors several viruses of the Flaviviridae family, including DENV, West Nile virus, Japanese encephalitis virus, and Yellow fever virus, but relatively little data are available regarding ZIKV. Cross-species reactions among members of the Flaviviridae family in Southeast Asia have limited the use of serologic assays, such as the hemagglutination inhibition, complement fixation, cross-neutralization, mouse protection, and hemagglutination assays (9). The first case of ZIKV infection in Southeast Asia was reported in the Philippines in 1953 (10). This was followed by cases identified and isolated in Malaysia in 1953 and 1954 (11), and Thailand and Vietnam in 1954 (10) by the cross-neutralization assay, Indonesia in 1977–1978 (12) and 1983 (13) by the hemagglutination inhibition assay, and Cambodia in 2010 (14) by reverse transcription–polymerase chain reaction (RT-PCR). However, the most commonly recommended assays for ZIKV detection include the plaque reduction neutralization test (PRNT), IgM antibody–capture enzyme-linked immunosorbent assay, and RT-PCR (15). Unfortunately, a considerable number of ZIKV infections throughout Southeast Asia may go unreported due to misclassification during detection. In addition, recent research in Latin America suggested that coinfections involving ZIKV, DENV, and Japanese encephalitis virus were more common than ZIKV infection alone because ZIKV has historically received little attention in Asian countries; thus, individuals who were first diagnosed with DENV or CHIKV infection using normal RT-PCR may not have been tested for ZIKV infection (16).

Southeast Asia has abundant natural habitats and forests that harbor an extensive diversity of wildlife. Nevertheless, human activities such as urbanization and movement patterns of both humans and non-human primates (NHPs) have led to increased encroachment on natural areas. Increased contact between humans, vectors, and wildlife can facilitate not only the spillover of diseases from NHPs to humans but also the spillback of human diseases from humans to wild primates. This close relationship has a number of negative effects, such as increased risk of disease transmission between humans and wildlife (17). The patterns of human behavior and changes in ecosystems have played an important role in the increasing emergence and re-emergence of infectious diseases (18). Pathogens can be spread from NHPs to humans via a broad range of transmission routes, including direct contact with bodily fluids, consumption of contaminated food or drink, and transmission via insect vectors (19, 20). As NHPs and humans have similar tissue architectures, immunological responses, physiological activities, and metabolic processes, they are frequently susceptible to the same infections. The recent emergence of the zoonotic disease COVID-19 became a global public health problem. As such, there is now more study and surveillance of emerging diseases that may be caused by infections transmitted from animals to humans (21).

Macaques (*Macaca* spp.) are among the most biodiverse animals, and they are widely distributed across a vast geographic range extending from North Africa to Asia (22). The extensive diversity and prevalence of macaques underscore their significance as a potential reservoir for arboviruses that infect humans. Several studies have investigated the seroprevalence of ZIKV infection in NHPs, and ZIKV seropositivity was identified in multiple species of NHPs, including both Old World and New World monkeys. Old World monkey exhibit a higher percentage of ZIKV seropositivity than the New World monkey (23). A previous study in Thailand examined the presence of neutralizing antibodies against ZIKV in wild stump-tailed macaques in a national park (24). The objective of this study was to determine the serum proteomic profile in wild stump-tailed macaque seropositive for antibodies against ZIKV. This comprehensive analysis of serum proteins provides insights into the various pathways activated within a host animal infected with ZIKV. It has been clearly established that cells or tissues that are infected or diseased often produce proteins or metabolic substances that can be detected in the serum. Therefore, studying the serum proteome of macaques could facilitate the identification of molecular responses and physiological changes that occur during ZIKV infection (25).

## 2 Methods

### 2.1 Ethics statement and sample collection

The study was conducted according to the applicable guidelines for the care and use of laboratory animals. The ethics committee of Kasetsart University Research and Development Institute at Kasetsart University, Thailand, approved all animal care and handling procedures (ID: ACKU67-SCI-006). The Thailand Department of National Parks, Wildlife, and Plant Conservation approved the study’s experimental methods for a conservation area, the collection of blood samples, and the release of wild macaques (permit number: 0909.204/14187).

A total of 32 blood samples were collected from wild stump-tailed macaques at the Pa La U waterfall, Huahin District, Prachuap Kiri Khan Province, Thailand in December 2018. The macaques were captured and immobilized via intramuscular injection with xylazine hydrochloride (0.5–2 mg/kg body weight) and tiletamine-zolazepam (2–5 mg/kg body weight). Blood samples were collected from the femoral vein and centrifuged at 2,200 × g for 20 min at 4°C to collect the serum, which was stored at −80°C. All macaques were physically examined by a veterinarian which no sign of illness was included in this experiment. Anthropological measurements were taken (weight, arm length, leg length, tail length, and body length), and gender was determined. Dental casts and dental photographs were taken.

### 2.2 Serology assessment

The 90% plaque reduction neutralization (PRNT90) assay was employed to detect neutralizing antibodies against ZIKV. This method determines the lowest serum dilution at which a 90% reduction in viral foci is observed, indicating the presence of neutralizing antibodies capable of inhibiting ZIKV infection. Test serum samples were diluted in OptiMEM (Invitrogen) serum diluent in a series of 4-fold dilutions starting at 1:5. Subsequently, the diluted serum samples were heat-inactivated at 56°C for 30 min. The serum diluent contained 0.3% human serum albumin. ZIKV was added to an equal volume of diluted serum and thoroughly mixed. The ZIKV had been previously diluted in serum diluent to achieve a final concentration of 1,000 plaque-forming units per milliliter. The virus/serum mixture was incubated at 37°C for 30 min, and cell growth medium was removed from 24-well plates containing Vero cell monolayers at approximately 90% confluence. Next, 50 μL of virus-serum combination was added to duplicate wells of the cell monolayers. Following a 60-min incubation at 37°C, the cell monolayers were overlaid with a solution consisting of 0.5% methylcellulose in OptiMEM supplemented with 2% fetal bovine serum. The samples were then cultured for 4 days to allow for ZIKV replication and plaque formation. Plaques were visualized using immunoperoxidase staining. Methylcellulose was subsequently removed from the infected monolayers, and the cells were fixed in 80% methanol for 30 min before washing with 5% nonfat milk in phosphate-buffered saline (PBS). Following 1:2,000 dilution in 5% nonfat milk, the monoclonal antibodies 2H2 (mouse anti–DENV1-4 prM protein) and 4G2 (mouse anti–Flavivirus envelope protein antibody, which binds to a conserved epitope E protein of the Flavivirus family) were added to each well. After allowing the fixed cell monolayers to sit at room temperature for 1 h, the primary antibodies were removed, and the cell monolayers were washed twice with PBS. Peroxidase-labeled goat anti-mouse IgG, diluted 1:2,000 in 5% nonfat milk, was then added to each well, and the plates were incubated for 1 h at 37°C. Following incubation, the wells were washed twice with PBS to remove the secondary antibodies. Subsequently, peroxidase substrate solution (4-chloro-1-naphthol in H_2_O_2_) was added to each well, and visible plaques were counted. The PRNT titer, indicating a 90% reduction in plaque count (PRNT90), was calculated using a curve-fitting method provided by the NIH/online NIAID tool.^1^ World Health Organization criteria were employed to differentiate ZIKV infection from infections involving other flaviviruses using PRNT90 antibody titers. According to these criteria (26, 27), ZIKV infection is defined as a PRNT90 titer ≥20, with a 4-fold difference observed between ZIKV and DENV PRNT90 titers.

### 2.3 Serum protein preparation and liquid chromatography–tandem mass spectrometry analysis

Serum protein content was determined using the Lowry protein assay method, with bovine serum albumin as the protein standard (28). To prepare the samples, 5 μg of serum protein was subjected to reduction of disulfide bonds using 5 mM dithiothreitol in 10 mM ammonium bicarbonate, followed by incubation at 60°C for 1 h. Subsequently, sulfhydryl group alkylation was carried out using 15 mM iodoacetamide in 10 mM ammonium bicarbonate at room temperature for 45 min in the dark. Carbamidomethylated proteins were then incubated with sequencing-grade trypsin at a ratio of 1:20, followed by overnight incubation at 37°C. The resulting tryptic peptides were dried using a speed vacuum concentrator, and the pellet was resuspended in 0.1% formic acid for nano LC–MS/MS analysis.

Tryptic peptides were injected into an Ultimate3000 Nano/Capillary LC System (Thermo Scientific, UK) coupled to an HCTUltra LC–MS system (Bruker Daltonics Ltd.; Hamburg, Germany) equipped with a Nano-captive spray ion source. Initially, 100 ng of peptide was enriched on a 300 μm ID × 5 mm C18 PepMap 100, 5 μm, 100 Å (Thermo Scientific, UK) column and then separated on a 75 μm ID × 15 cm column packed with Acclaim PepMap RSLC C18, 2 μm, 100, nanoViper (Thermo Scientific, UK). The C18 column was maintained in a column oven set at 60°C. Solvents A and B, consisting of 0.1% formic acid in water and 0.1% formic acid in 80% acetonitrile, respectively, were employed for chromatographic separation. Peptides were eluted over 30 min at a constant flow rate of 0.30 μL/min using a gradient of solvent B ranging from 5 to 55%. Electrospray ionization was conducted at 1.6 kV using CaptiveSpray, with nitrogen gas at a flow rate of approximately 50 L/h as the drying gas. Collision-induced dissociation product ion mass spectra were acquired with nitrogen gas as the collision gas. Mass spectra were collected in positive-ion mode over a mass range of 150 to 2,200 *m/z* at a frequency of 2 Hz. The collision energy was adjusted to 10 eV based on the *m/z* value. Each sample was subjected to triple LC–MS analysis for robust data acquisition. Each sample was subjected to triple LC–MS analysis for robust data acquisition.

### 2.4 Protein identification and quantification

LC–MS/MS data were analyzed for protein quantification using DeCyder MS Differential Analysis software. Proteins were identified by searching the UniProt Macaca protein database utilizing the Mascot search engine. Trypsin was employed as the digesting enzyme, and the standard settings in Mascot included a maximum of three missed tryptic cleavages, a fragment peptide mass tolerance of 1.2 Da, and an MS/MS tolerance of 0.6 Da. Additionally, cysteine carbamidomethylation was selected as the fixed modification, whereas methionine oxidation was selected as the variable modification. Peptide charge states of 1+, 2+, and 3+ were included. Protein expression levels (PELs) derived from MS/MS spectra are expressed as log2 values.

### 2.5 Data analysis

ZIKV-neutralizing antibodies were detected using PRNT90 assays. Statistical analysis was conducted using the Mann–Whitney *U* test to compare the naïve and seropositive groups. IBM SPSS Statistics version 22.0.0 was employed for this analysis, utilizing PEL values as input data. A significance threshold of >95% was set for the *p-*value cutoff, with values below this threshold considered statistically significant.

Proteins were functionally categorized using the EuKaryotic Orthologous Groups (KOGs)^2^ and KEGG Orthologous (KO)^3^ databases. The KEGG Orthology And Links Annotation (GhostKOALA).^4^ The unknown proteins were fulfilled using the Protein Basic Local Alignment Search Tool (BLAST P).^5^ The Protein Analysis Through Evolutionary Relationships (i.e., PANTHER)^6^ classification system was employed to describe cellular functional gene ontology. Functional protein association networks were generated using the STITCH^7^ and STRING^8^ databases, predicting and constructing protein-chemical and protein–protein interaction networks with a high confidence score. The networks were visualized using Cytoscape, version 3.9.0, leveraging data from the STITCH and STRING databases.

### 2.6 Supervised and unsupervised machine learning analysis

The three protein subsets include 9,532 total proteins, 338 DEPs, and 52 important proteins that were used to supervise machine analysis. The five algorithms were used to prediction classification: support vector machine (SVM), k-nearest neighbors (KNN), logistic regression (LR), random forest (RF), and multinomial naïve Bayes (MNB) classifier. The polynomial kernel of degree two as parameters of support vector machine algorithm (29, 30). The number of neighbors (K) was run with three, and the Euclidean distance metric on the KNN model algorithm (29, 30). LR algorithm uses parameters such as a logistic function to model a binary output variable and the Broydene Fletcher Gold Farbe Shanno algorithm has been used as an iterative method for solving unconstrained nonlinear optimization problems (29). RF, the number of trees in the forest used was 1,000, the supported criteria were Gini impurity. The maximum depth of the tree was none, minimum sample split, and minimum sample leaf as 4 and 2, respectively (29). The MNB classifier is appropriate for classification tasks involving discrete features. For the current analysis, the parameter alpha was configured to 0.1 in this model (31). The accuracy of the predicted classifications for the samples was determined using the leave-one-out cross-validation (LOOCV) algorithm.

The unsupervised principal component analysis was used to compare the dimensions of feature proteins. For sample group clustering, the 32 samples were categorized into 2 primary clusters: seropositive and naïve. Samples in each cluster were examined by unsupervised hierarchical clustering analysis based on relative PELs.

## 3 Results

### 3.1 Detection of neutralizing antibodies against ZIKV

**Figure 1:**
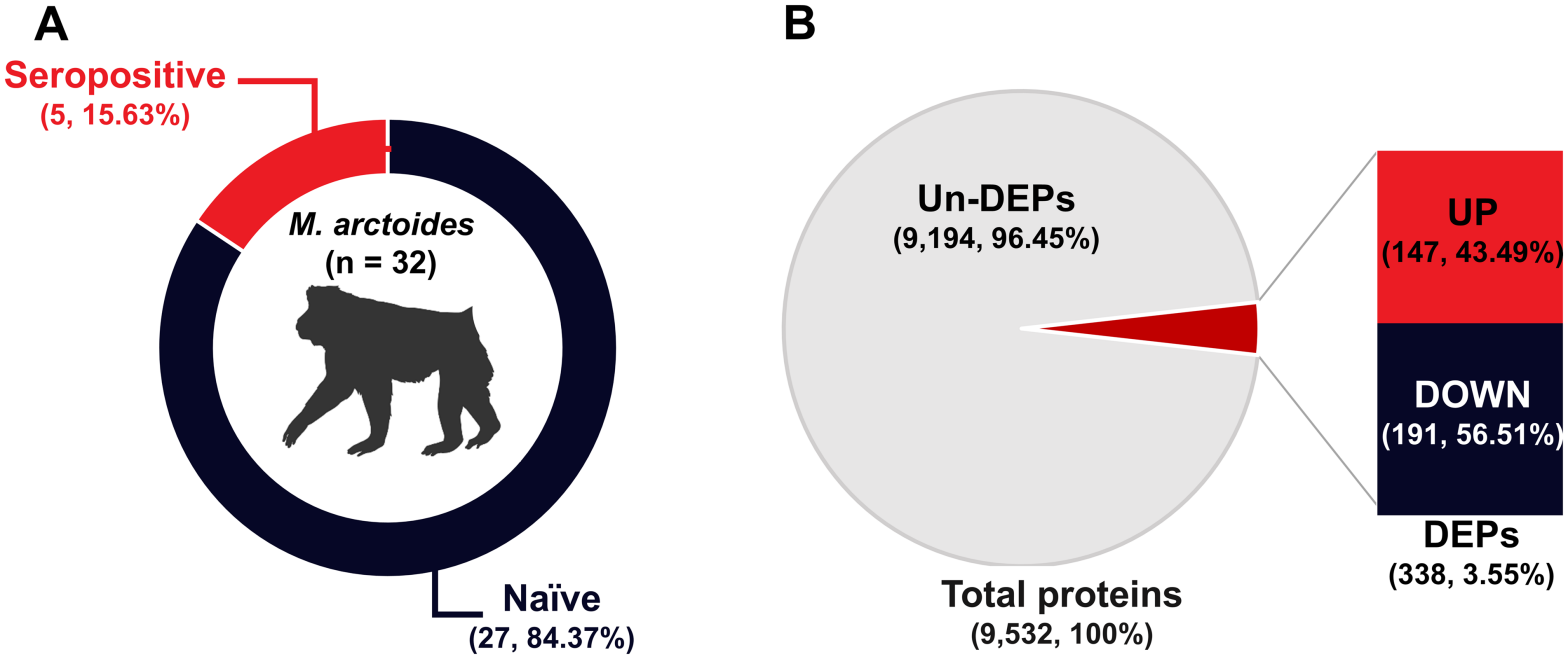
Simian ZIKV antibody detection and protein identification. (A) Percentage of animals in the naïve and seropositive groups. (B) The pie chart shows the total proteins, whereas the bar chart indicates the up- and down-regulation of DEPs.

Samples collected from 32 wild stump-tailed macaques at the Pa La U waterfall in the Hua Hin District, Prachuap Khiri Khan Province, Thailand, were subjected to PRNT90 assays against ZIKV. Seropositivity was defined as a PRNT90 titer >1:20 with a 4-fold difference observed between ZIKV and DENV PRNT90 titers. A total of 5 samples (15.63%) tested positive for ZIKV, whereas 27 samples (84.37%) tested negative (Figure 1A). These samples were then divided into two groups: seropositive and naïve.

### 3.2 Biological function enrichment of proteins

Based on correlations between MS/MS spectra and *Macaca* proteins in the UniProt database, we identified a total of 9,532 unique proteins in serum samples from the 32 animals. A total of 3,707 proteins (38.89%) were up-regulated in expression, whereas 5,825 proteins (61.11%) were down-regulated. Furthermore, a total of 338 differentially expressed proteins (DEPs) were identified. Of these DEPs, 147 (43.49%) were up-regulated in expression, and 191 (56.51%) were down-regulated (Figure 1B).

**Figure 2:**
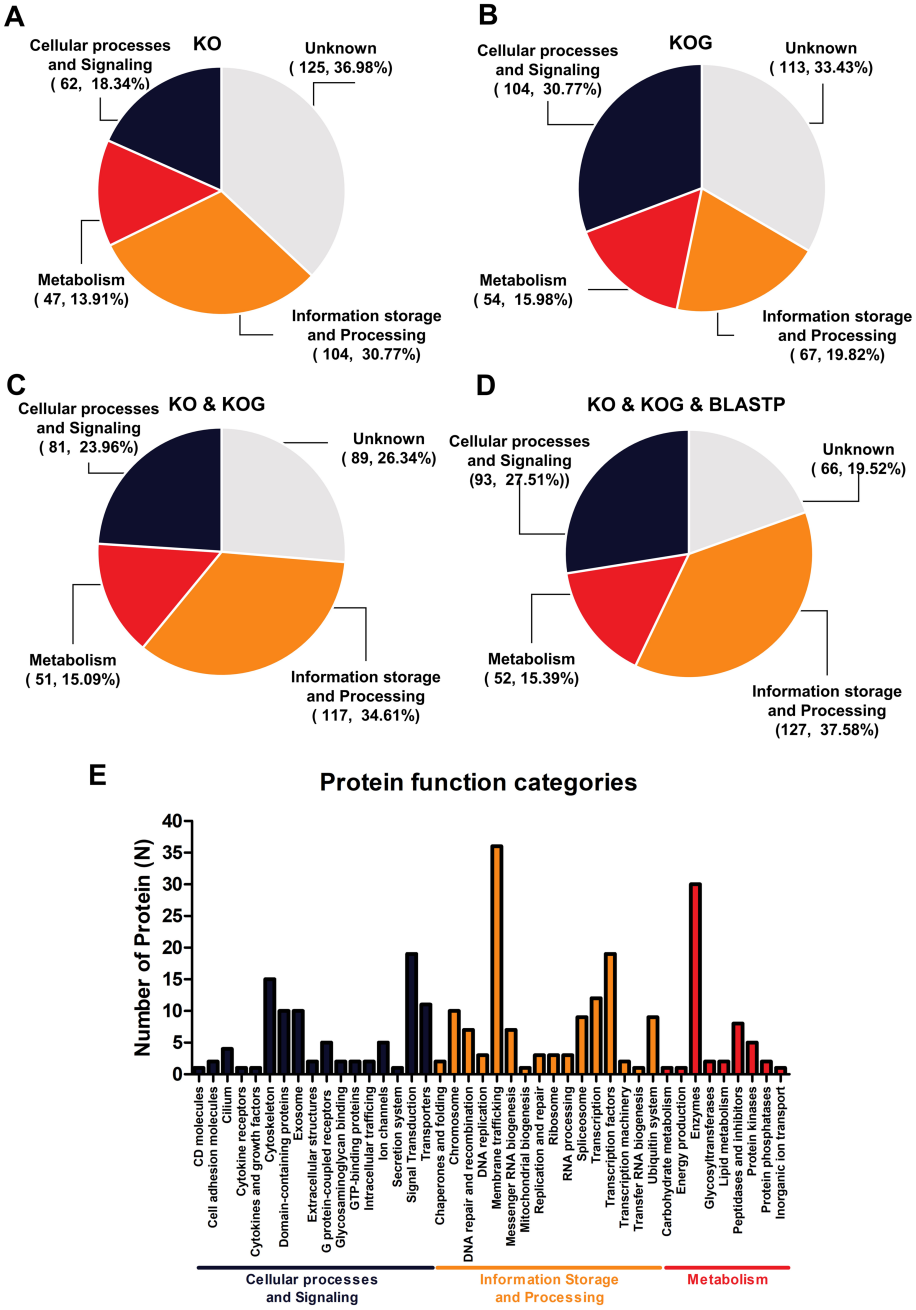
Enrichment of orthologous genes in terms of biological functions for 338 proteins. Gene orthologous analysis was used to categorize (A) the number of proteins identified using the KO database and (B) the number identified using the KOGs database, whereas (C) shows the combined results for the KO and KOGs databases. (D) Shows the combined results for the KO and KOGs and BLASTP databases and descriptive function of classifications (E).

**Figure 3:**
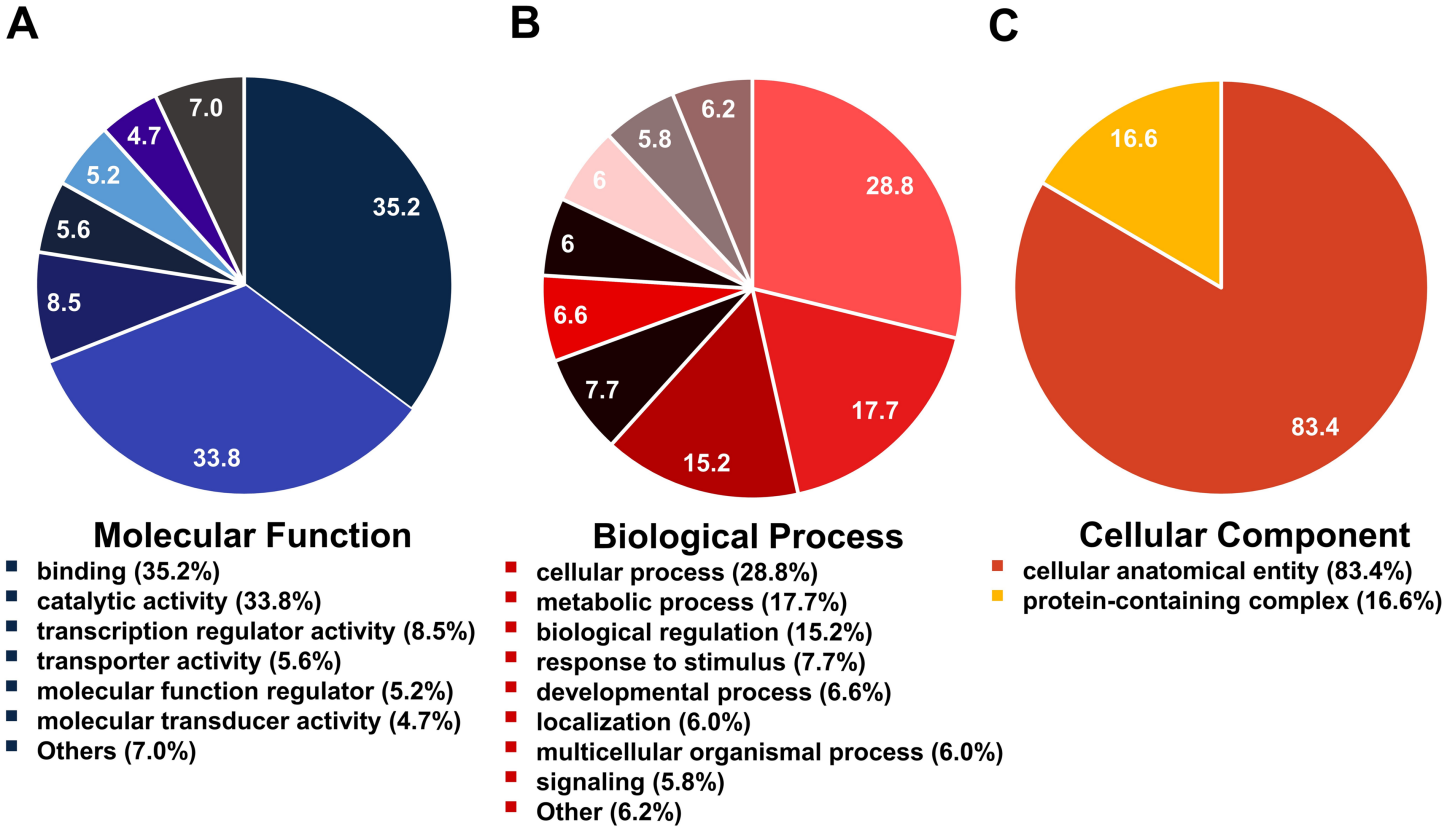
Biological function enrichment gene ontology analysis based on 338 proteins. Classification based on gene ontology regarding (A) molecular function, (B) biological process, and (C) cellular component.

A serum proteome profile related to ZIKV seropositivity was constructed using gene orthologous categorization. The KO database was used to identify the functions of DEPs and revealed approximately 213 unique proteins (63.02%). These proteins were predominantly associated with information storage and processing (104, 30.77%), followed by cellular processes and signaling (62, 18.34%) and metabolism (47, 13.91%) (Figure 2A). Conversely, annotation using the KOG database revealed that the highest number of proteins was in the category of cellular processes and signaling (104, 30.77%), followed by information storage and processing (67, 19.82%) and metabolism (54, 15.98%) (Figure 2B). However, the two databases describe orthologous genes differently, as the KOG database was often used to describe comparative genomics and evolutionary studies of eukaryotic organisms, while the KO database tends to focus on the functional role in pathways. Although the protein identifications of the two databases were grouped differently, for ease of study, the two databases were combined to complement proteins with unknown functions. Moreover, when the two databases were combined, the KO database served as the primary database because it focused on the functional role of pathways. A notable increase in the number of functionally unique proteins (73.67%) was observed. This included proteins primarily associated with cellular processes and signaling (115, 34.02%), followed by information storage and processing (77, 22.78%) and metabolism (57, 16.86%) (Figure 2C). Moreover, the 89 unknown proteins were re-annotated with BLAST P, and the 22 proteins were filled with annotation gene orthologous. Overall, this gene orthologous profile includes cellular processes and signaling (126, 37.28%), followed by information storage and processing (87, 25.74%) and metabolism (58, 17.16%) as indicated in Figure 2D. Detailed analyses by the KO, KOG, and BLASTP databases indicated that most of the proteins were related to membrane trafficking (information storage and processing) estimated as 10.06% from 338 proteins, followed by 8.88% enzymes (metabolism), 5.62% transcription factors (information storage and processing), 4.14% cytoskeleton (cellular processes and signaling), and others as indicated in Figure 2E. To further enrich the serum proteome analysis, gene ontology analysis was carried out to determine the diverse roles of the identified proteins in various biological functions, cellular processes, and molecular activities in the serum proteome profile related to ZIKV antibody seropositivity in wild stump-tailed macaques. In the categorization based on molecular function, the majority of proteins were associated with binding (35.2%), followed by catalytic activity (33.8%), transcription regulator activity (8.5%), and other minor functions (22.5%) (Figure 3A). Regarding biological processes, the functions were distributed as follows: cellular processes (28.8%), metabolic processes (17.7%), biological regulation (15.2%), response to stimulus (7.7%), and other minor functions (30.6%) (Figure 3B). Furthermore, in terms of cellular components, the identified proteins were mainly related to cellular anatomical entities (83.4%), with a smaller proportion associated with protein-containing complexes (16.6%) (Figure 3C).

### 3.3 Protein interaction networks

**Figure 4:**
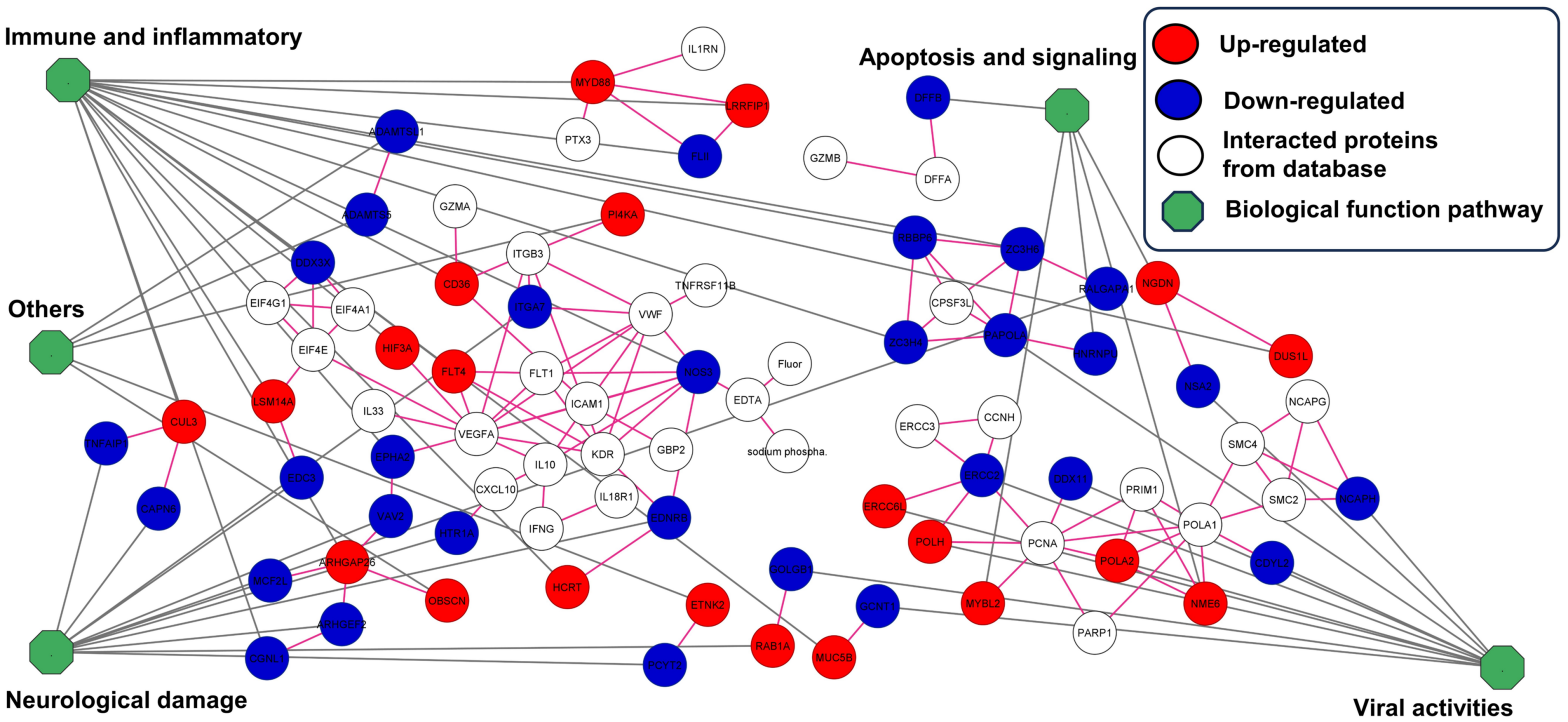
Construction of protein interaction networks for 52 important proteins in the seropositive group. Proteins were classified into five pathways. In the network visualization, proteins exhibiting up-regulated expression levels are indicated in red, whereas those exhibiting down-regulated expression levels are indicated in blue. Additionally, white indicates interacting proteins from the database, and the green octagon indicates biological pathways.

STITCH and STRING were used to establish protein interaction networks for the 338 DEPs and illustrate the involvement of the identified proteins in various biological processes and pathways associated with ZIKV infection in wild stump-tailed macaques. This led to the identification of 52 important proteins. These 52 proteins were classified into five pathways based on function. The predominant pathway was immune and inflammatory processes (36.53%), followed by pathways associated with neurological damage (23.08%), viral activities (21.15%), apoptosis signaling (9.61%), and other pathways (9.61%). The distribution of proteins across the different pathways is illustrated in Figure 4 and summarized in Table 1.

**Table 1:** Characteristics of 52 important proteins and biological function pathways.

Gene name	Protein name	*p*-value	FC	% (Naïve)	% (Positive)	Expression
**Apoptosis signaling pathway**						
*DFFB*	DNA fragmentation factor subunit beta	0.018	0.95	100	100	Down
*HNRNPU*	Heterogeneous nuclear ribonucleoprotein U isoform b	0.019	0	81.48	40	Down
*MYBL2*	MYB proto-oncogene like 2	0.014	16.42	18.52	80	UP
*NGDN*	Neuroguidin isoform 2	0.03	1.05	74.07	100	UP
*NME6*	Nucleoside diphosphate kinase	0.029	16.76	14.81	60	UP
**Viral activities**						
*CDYL2*	Chromo domain-containing protein	0.035	0	55.56	0	Down
*CGNL1*	Cingulin like 1	0.035	0	70.37	20	Down
*DDX11*	Helicase ATP-binding domain–containing protein	0.026	0	59.26	0	Down
*ERCC2*	General transcription and DNA repair factor IIH helicase subunit XPD	0.037	0	77.78	20	Down
*GOLGB1*	Golgin B1	0.007	0	92.59	40	Down
*NCAPH*	Condensin complex subunit 2	0.042	0	62.96	20	Down
*NSA2*	Ribosome biogenesis protein NSA2 homolog	0.014	0	74.07	20	Down
*PAPOLA*	Poly(A) polymerase	0.045	0	51.85	0	Down
*ERCC6L*	ERCC excision repair 6 like, spindle assembly checkpoint helicase	0.026	1.12	51.85	100	UP
*POLA2*	DNA polymerase alpha subunit B	0.018	1.13	55.56	100	UP
*POLH*	DNA polymerase eta	0.033	15.51	40.74	100	UP
**Neurological damage**						
*ARHGEF2*	Rho/Rac guanine nucleotide exchange factor 2	0.042	0	62.96	20	Down
*CAPN6*	Calpain-6 (Fragment)	0.02	0	77.78	40	Down
*EDC3*	Enhancer of mRNA-decapping protein 3	0.02	0	0	20	Down
*EDNRB*	Endothelin receptor type B	0.016	0	3.7	40	Down
*HTR1A*	5-Hydroxytryptamine receptor 1A	0.045	0	51.85	0	Down
*ITGA7*	Integrin subunit alpha 7	0.042	0	62.96	20	Down
*MCF2L*	MCF.2 cell line–derived transforming sequence like	0.045	0	51.85	0	Down
*PCYT2*	Ethanolamine-phosphate cytidylyltransferase isoform 2	0.025	0	81.48	40	Down
*RALGAPA1*	Rap-GAP domain-containing protein	0.018	0.94	96.3	100	Down
*TNFAIP1*	TNF-alpha–induced protein 1	0.031	0	74.07	40	Down
*VAV2*	Vav guanine nucleotide exchange factor 2	0.045	0	51.85	0	Down
*RAB1A*	RAB1A, member RAS onco family	0.008	12.67	7.41	60	UP
**Immune and inflammatory responses**						
*DDX3X*	RNA helicase	0.045	0.95	88.89	60	Down
*EPHA2*	Ephrin type-A receptor 2	0.031	0.96	92.59	60	Down
*FLII*	FLII actin remodeling protein	0.016	0.91	92.59	60	Down
*GCNT1*	Glucosaminyl (N-acetyl) transferase 1	0.013	0	85.19	40	Down
*NOS3*	Nitric oxide synthase	0.045	0.92	81.48	60	Down
*RBBP6*	E3 ubiquitin-protein ligase RBBP6	0.01	0.92	92.59	80	Down
*ZC3H4*	Zinc finger CCCH-type containing 4	0.041	0	74.07	20	Down
*ZC3H6*	Zinc finger CCCH-type containing 6	0.015	0	66.67	0	Down
*ARHGAP26*	Rho GTPase–activating protein 26	0.01	1.07	92.59	100	UP
*CD36*	Platelet glycoprotein 4	0.001	16.93	11.11	80	UP
*CUL3*	Cullin-3	0.022	1.04	62.96	100	UP
*DUS1L*	Dihydrouridine synthase 1 like	0.004	1.13	66.67	100	UP
*FLT4*	Receptor protein-tyrosine kinase	0.031	1.06	81.48	100	UP
*HCRT*	Orexin (Hypocretin)	0.018	1.24	81.48	100	UP
*HIF3A*	Hypoxia-inducible factor 3 subunit alpha	0.046	14.74	40.74	80	UP
*LRRFIP1*	Leucine-rich repeat flightless-interacting protein 1 isoform 5	0.036	1.09	100	100	UP
*LSM14A*	LSM14A mRNA processing body assembly factor	0.014	1.23	81.48	100	UP
*MUC5B*	Mucin 5B, oligomeric mucus/gel-forming	0.047	11.93	25.93	60	UP
*MYD88*	Myeloid differentiation primary response protein MyD88	0.035	1.06	88.89	100	UP
**Others**						
*ADAMTS5*	Peptidase M12B domain-containing protein	0.045	0	51.85	0	Down
*ADAMTSL1*	ADAMTS like 1	0.02	0	0	20	Down
*PI4KA*	1-Phosphatidylinositol 4-kinase	0.02	1.09	70.37	100	UP
*ETNK2*	Ethanolamine kinase 2	0.02	1.27	55.56	100	UP
*OBSCN*	Obscurin	0.006	18.52	25.93	80	UP

% (Naïve) = (number of proteins detected in naïve samples/number in naïve samples) × 100; % (Positive) = (number of proteins detected in seropositive samples/number in seropositive samples) × 100 FoldChange (FC) = PELs (seropositive)/PELs (naïve).

### 3.4 Supervised and unsupervised machine learning for support profile

The three protein subsets were compared in the percentage of accuracy score in Table 2. The 9,532 features of total proteins represented 84.38% in five algorithms, while when the feature proteins were selected by statistical analysis as 338 features of DEPs. The SVM, KNN, LR, and algorithms increased their accuracy score from 84.38 to 100%, and MBN increased their accuracy score from 84.38 to 96.88%. However, RF had the same accuracy score between total protein and DEPs. Interestingly, the 52 features of important proteins represented the highest accuracy score in all five algorithms. The four algorithms SVM, KNN, LR, and MNB were predicted to have 100% accuracy, while RF was expected to have an 87.50% accuracy score. However, the important proteins showed the highest accuracy compared to other protein subsets. However, a perfect accuracy score of 100% can indicate that the 52 proteins are rich in protein features that are important for disease prediction, which often raises the suspicion of overfitting. It essentially means the model has learned the noise and specific patterns of the training set rather than the underlying distribution of the data, leading to artificially high accuracy. Ultimately, the purpose of using machine learning to analyze is simply to use it to support and compare the datasets.

**Table 2:** Supervised machine learning classification algorithm for support profile.

Protein subsets	Total proteins	DEPS	Important proteins
Number of features	9,532 features	338 features	52 features
Classification algorithm	Accuracy	Accuracy	Accuracy
Support vector machine	84.38%	100.00%*	100.00%*
K-nearest neighbors	84.38%	100.00%*	100.00%*
Random forest	84.38%	84.38%	87.50%*
Logistic regression	84.38%	100.00%*	100.00%*
Multinomial naïve Bayes	84.38%	96.88%	100.00%*

*Highest accuracy score.

**Figure 5:**
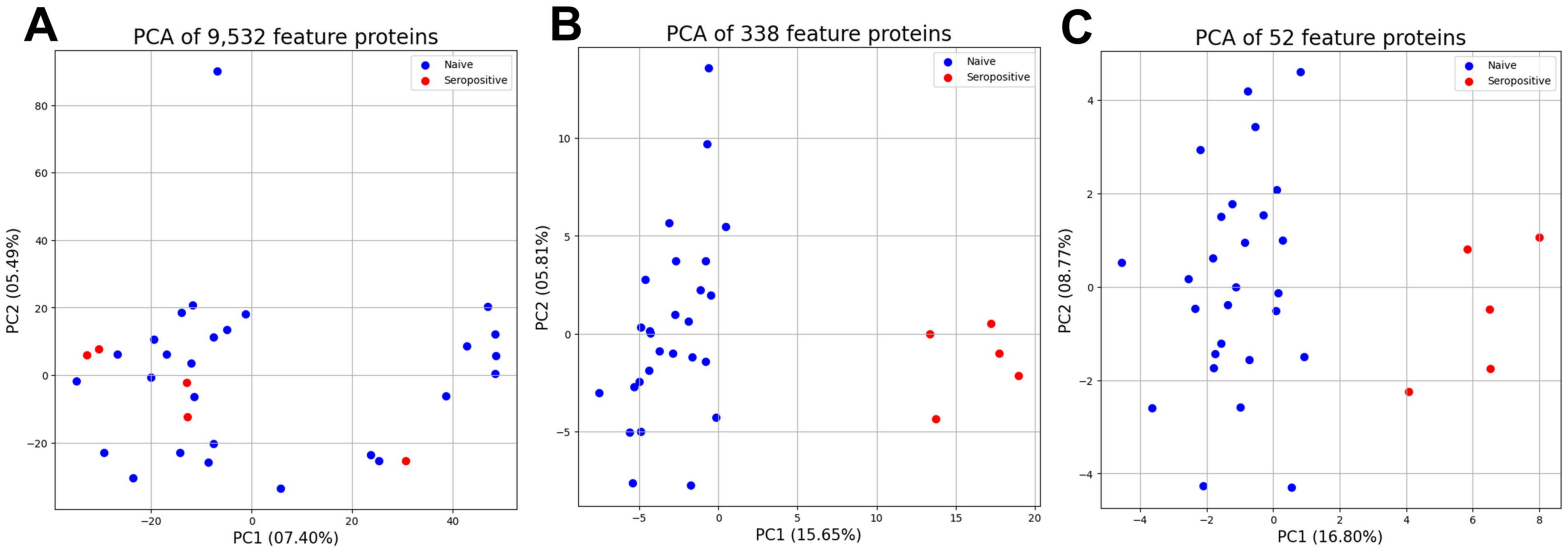
Unsupervised principal component analysis between three proteins subset for support profiling. The principal component analysis of 9,532 feature proteins (A), and 338 differentially feature proteins (B), and 52 important feature proteins (C).

The three protein subsets were used to compare dimensions by unsupervised principal component analysis (Figure 5). The PCA score of 9,532 features proteins was 12.89% including PC1 (7.40%) and PC2 (5.49) (Figure 5A). However, the 338 feature proteins as proteins after statistical analysis represented the increased PCA score of 21.46% including PC1 (15.65%) and PC2 (5.81%) (Figure 5B). Moreover, the 52 feature proteins as proteins after interaction analysis represented the highest PCA score at 25.57% including PC1 (16.80%) and PC2 (8.77%) (Figure 5C). Both supervised classification prediction machine learning and unsupervised principal component analysis highlighted that the 52 feature proteins were the most promising dataset for protein profiling.

**Figure 6:**
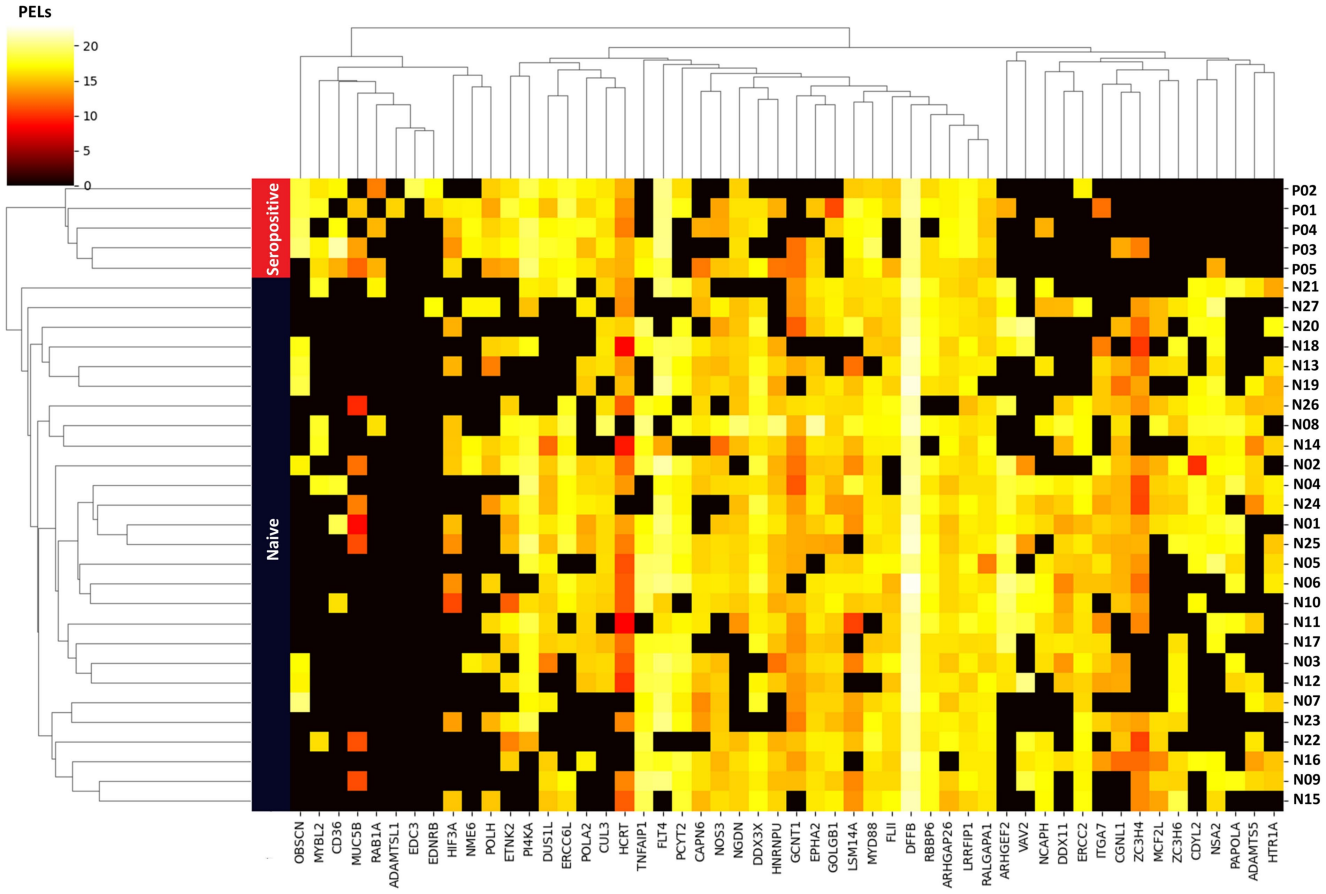
Unsupervised hierarchical clustering heatmap analysis based on 52 important proteins as a feature. Vertical rows represent the 32 samples, with blue and red bands indicating samples from the naïve and seropositive groups, respectively. The 52 proteins are listed on the horizontal axis. The color gradient within the heatmap reflects the scale of protein expression level (PEL) for each sample, with colors indicating the range from low to high expression level.

Unsupervised hierarchical clustering analysis of the 52 important proteins revealed distinct patterns of protein expression that differentiated and enabled clear clustering of the seropositive and naïve groups. In the generated plot (Figure 6), the y-axis shows the PEL of the 52 important proteins, whereas the X-axis depicts the 32 samples.

## 4 Discussion

Proteomics is one of the most popular approaches for studying proteins because it provides an overview of proteins in terms of function and structure, as well as protein–protein and protein-chemical interactions. In this study, we used a proteomics approach to study the serum proteins of wild macaques in which antibodies against ZIKV were detected. Bioinformatics analysis of the resulting LC–MS/MS data led to the identification of 52 important proteins. These proteins were categorized into five pathways: apoptosis signaling, viral activities, neurological damage, immune and inflammation responses, and others.

### 4.1 Apoptosis signaling pathway

Apoptosis, or programmed cell death, is a host defense mechanism that prevents viral transmission by eliminating infected cells, thereby inhibiting viral takeover of cellular metabolism and nucleotide and protein synthesis without affecting non-infected cells while inducing antigen-presenting and immune cells (32). In general, viral infections induce pro-apoptosis signaling pathways. Some viruses are activated via intrinsic pathways, whereas others are activated via extrinsic pathways (33). Several studies have indicated that ZIKV infection tends to differentially inhibit apoptosis signaling pathways to enable the virus to contend for cellular resources. Wu et al. (34) reported that in human epithelial cells derived from fibrosarcoma infected with ZIKV, activation of the apoptotic pathway is delayed by the ZIKV NS2B/3 proteins, which inhibit the JAK–STAT pathway. Turpin et al. (35) reported that ZIKV suppresses the apoptosis signaling pathway in infected cells and protects against exogenous apoptosis generated by either intrinsic or extrinsic pathways in cells derived from human malignant glioblastoma and in human embryonic kidney 293 cells. Muthuraj et al. (36) reported that apoptosis is inhibited in TR-8, JEG-3, and JAR cells, CHOP is overexpressed and nuclear translocation is discovered. The use of JNK inhibitors significantly reduces apoptosis. However, our study identified five proteins related to apoptosis signaling pathways, and we found that two proteins decreased apoptosis signaling pathway activity, and three proteins increased the activity of the anti-apoptosis pathway. HNRNPU is a member of the HnRNP family of proteins that exert RNA-related metabolic activities, such as pre-mRNA splicing, transcription, and translation control. In the apoptosis pathway, HNRNPU is a limiting factor for anti-apoptosis activity (caspase-9b) regulated by RNA trans-factors associated with exon 3 of caspase-9 pre-mRNA (C9/E3) (37). DFF (DNA fragmentation factor) is a protein that plays a crucial role in the process of apoptosis. It is a heterodimeric protein consisting of two subunits, DFFA and DFFB. DFFA serves as a substrate for caspase-3, a key enzyme in apoptosis. Caspase-3 cleaves and activates DFFA, initiating a cascade of events that ultimately lead to DNA fragmentation. This process is a hallmark of apoptosis, as it allows for the orderly and controlled breakdown of cellular genetic material. In contrast, DFFB, as identified in our study, plays a dual role in apoptosis. DFFB triggers both DNA fragmentation and chromatin condensation. Chromatin condensation involves the compaction of DNA within the cell nucleus, further contributing to the orderly dismantling of cells during apoptosis (38).

Our study found that two down-regulated proteins are involved in increasing anti-apoptosis pathway activity and decreased apoptosis. Neuroguidin interacts with components of the survival motor neuron (SMN) complex. Neuroguidin is an eIF4E-interacting protein essential for cytoplasmic polyadenylation element binding protein-mediated translation of important genes during early development and for synaptic plasticity in neurons. The SMN complex is involved in splicing, neuronal migration, and differentiation (39). The SMN protein is the predominant factor responsible for spinal muscular atrophy, a hereditary neurodegenerative disorder that manifests in childhood (40). However, SMN overexpression in SH-SY5Y cells confers protection against Akt/PI3-kinase inhibition in rat cortical neurons, but not against oxidative stress or excitotoxicity. Further exploration through Western blotting analysis revealed a notable anti-apoptotic function of SMN, specifically inhibiting caspase-3 activation by impeding calpain-mediated procaspase-3 cleavage. This discovery unveiled a novel facet of the cellular function of SMN in differentiated SH-SY5Y cells, emphasizing its capacity to inhibit apoptosis (41). In addition, NME6 is reportedly involved in the production of nucleoside triphosphates other than ATP, and it is also an inhibitor of p53-induced apoptosis. Deng et al. (42) examined the function of MYBL2 and reported that increased MYBL2 expression promotes synergy with CDC20 to inhibit apoptosis via the Wnt/*β*-catenin signaling pathway in gastric cancer. MYBL2 also reportedly regulates the expression of apolipoprotein J/clusterin and directly mediates resistance to apoptosis induction (43). Our study profiled proteins involved in the apoptosis signaling pathway in the serum of macaques in which ZIKV antibodies were detected. Five of the identified proteins are likely involved in inhibiting apoptosis.

### 4.2 Viral activities

One of the key mechanisms by which viruses evade the host immune system involves suppression of protein synthesis. Viruses disrupt cell metabolism, ultimately leading to cell death. Therefore, RNA viruses induce ribosomes to interrupt host protein translation, inhibit host mRNA recognition, and prevent ribosome subunit assembly. In our study, we identified eight proteins that decreased regulation of ribosome biogenesis, DNA replication, and DNA activity. NSA2 ribosome biogenesis protein is involved in formation of the 60S ribosomal subunit. It may play an essential role in the quality control of pre-60S particle formation (44). PAPOLA, a polymerase responsible for mRNA 3′-poly(A) tail synthesis, is critical for the post-transcriptional processing of RNA molecules. ERCC2 and DDX11 are involved in DNA replication, DNA repair, and heterochromatin organization, as well as in ribosomal RNA synthesis. CDYL2, NCAPH involved in DNA activity, the development of viral replication organelles is a complex process involving the cell membrane, with the endoplasmic reticulum being the primary target. In addition, decreased expression of these proteins demonstrates host resistance to competition for intracellular resources and inhibits viral multiplication (45). The formation of intercisternal substances in the Golgi complex is down-regulated through GOLGB1. CGNL1 is associated with tight junctions in actin-based cytoskeletons.

In contrast, we identified three proteins that exhibited increased expression; these proteins were involved in DNA damage, repair, and translocation. The complex relationship between DNA viruses and host DNA damage and repair machinery illustrates the complex processes exhibited by viruses that ensure successful replication and proliferation. Extensive research has revealed that the replicative life cycles of many DNA viruses involve complex interactions with physiological components involved in the DNA damage response and repair. DNA viruses selectively rely upon or disrupt specific parts of the cellular replication and repair mechanism by “hijacking” and manipulating essential components of the cellular replication and repair machinery (46). Here, we identified three overexpressed proteins: POLA2 and POLH as DNA polymerases and ERCC6L as a DNA translocase.

### 4.3 Neurological damage

The unique interaction profile with cellular components involved in neuronal development or neurological disorders distinguishes the capsule of ZIKV from that of other flaviviruses. Although many flavivirus capsids recruit proteins, the ZIKV capsid is unique in that it is associated with the neurological system (39). ARHGEF2 is expressed in structures such as the brain and dorsal root ganglia and enhances guanyl-nucleotide exchange factor and microtubule binding activity, thereby assisting in the development of mitotic spindle orientation, neuron production, and the positive regulation of neuron migration (47, 48). ARHGEF2 expression was found to be decreased in our study, which could indicate damage to neurons.

The MCF2L gene encodes a guanine nucleotide exchange factor that interacts specifically with GTP-bound Rac1 as well as Vav guanine nucleotide exchange factors (VAV), which have been implicated in cell adhesion involving integrins and immune response receptors via Rho GTPase regulation. These proteins play an important role in antigen receptor–mediated stimulation of T and B lymphocytes. Furthermore, decreased VAV expression inhibits immunological responses to the nonrepetitive T cell–dependent hapten antigen (4-hydroxy-5-iodo-3-nitrophenyl)acetyl (NIP)-OVA (49).

EDNRB is an important housekeeping factor for appropriate myelin sheath regulation, and it promotes reactive astrogliosis and assists in brain injury healing. EDNRB expression is down-regulated in both ZIKV-infected mouse primary astrocytes and U251 astrocytes, revealing a potential malfunction associated with ZIKV-induced neurological diseases (50). The specific roles of ITGA7 (*α*7β1) integrins as laminin-binding receptors are crucial for axonal sorting. Schwann cells utilize lamellipodia-like processes to segregate large- and small-caliber axons, a crucial step for subsequent myelination. Deficiency of ITGA7 adversely affects Schwann cell spreading and laminin-binding, which provides a potential explanation for the impaired extension of cytoplasmic processes around axons and hindering of the sorting process (51).

TNFAIP1 was identified as a tumor necrosis factor-α (TNF-α)–inducible protein with involvement in various cellular processes such as the cytokinesis signaling pathway, DNA synthesis, innate immunity, apoptosis, and it is believed to be relevant to diseases such as Alzheimer’s disease (52) The role of TNFAIP1 in the development of cortical neurons and its impact on neurological functions are likely to play a crucial role in human brain development and disease. Furthermore, TNFAIP1 contributes to the induction of neurotoxicity by negatively affecting the Akt/CREB signaling pathway, downregulating Bcl-2 expression, and inhibiting the transcriptional activity of nuclear factor–kappa B (NF-κB).

Ral (Ras-like) GTPases play an essential role in cell migration regulation and interact with RalA and RalB. Wagner et al. (53) reported increased RalA activity in various cell lines, confirming the hypothesis that reduced RalGAPA1 expression increases RalA constitutive activation. Furthermore, RalGAPA1 elimination significantly increases cell-surface levels of lipid raft components in detached fibroblasts, suggesting that anchorage-dependent cell growth signaling is disrupted. Disruption of the RalA pathway has a significant impact on neuronal function and brain development. Given the somewhat overlapping phenotypes of RALA- and RALGAPA1-associated disorders, it appears plausible that disruption of the RalA signaling pathway results in a new category of hereditary syndromes that we propose calling “RALopathies.”

The major enzyme regulating the *de novo* production of phosphatidylethanolamine (PE) via the CDP-ethanolamine Kennedy path is ethanolamine-phosphate cytidyltransferase 2. PE is an important lipid in cellular membranes, especially in the protoplasmatic leaflet. It plays an important role in a variety of cellular activities, such as membrane fusion, cell cycle control, autophagy, and apoptosis (54). Leonardis et al. (55) reported that the emergence of axonal motor and sensory polyneuropathy is related to ethanolamine-phosphate cytidyltransferase 2. The 5-hydroxytryptamine receptor 1A, also known as serotonin 1A receptor, is a subtype of serotonin receptors, or 5-hydroxytryptophan receptors that bind serotonin, a neurotransmitter. Ling and Raikhel (56) discovered an important relationship between mosquito blood feeding and serotonin concentration changes, specifically through the serotonin receptor Aa5HT2B in the fat-body.

Enhancer of mRNA-decapping protein 3 (EDC3) is involved in mRNA degradation and the positive control of mRNA decapping. Ahmed et al. (57) reported that abnormalities in this gene affect neurological diseases. EDC3 interacts with and promotes DCP2. A mutation was discovered in EDC3 in the LSm domain, which is involved in the interaction, indicating that this engagement could have been disrupted. Functional investigations revealed that mutated EDC3 not only fails to increase DCP2 activity but actively inhibits DCP2 function at high concentrations. Mutated EDC3 exhibits reduced efficacy in triggering DCP2 decapping in affected individuals, resulting in abnormal accumulation of a subset of mRNAs. This dysregulation is thought to have a detrimental effect on normal brain function and may be a contributing factor in intellectual disabilities in affected populations.

Brain seizures and brain damage are prevalent neurological symptoms of viral encephalitis, which are a major global health concern, particularly among children. Hippocampal injury leads to CAPN6 activation after acute picornavirus infection in mice, leading to neuronal death caused by invading inflammatory monocytes in the brain, according to researchers. According to one study, CAPN6 inhibitors may protect neurons against “bystander” harm resulting from immunological responses (58).

Rab1 GTPases are essential regulators of Golgi dynamics and membrane integrity, regulating bidirectional transport between the endoplasmic reticulum and the Golgi apparatus, as well as Golgi membrane synthesis, maintenance, and recycling. The complex regulation of Rab1 GTPases and their impact on Golgi dynamics, tau secretion in Alzheimer’s disease, and motor function in Parkinson’s disease underscore the therapeutic promise of targeting Rab1A in neurodegenerative processes. Rab1A as a therapeutic target could lead to new treatments for illnesses defined by disturbed cellular membrane fluidity and protein aggregation accumulation in the nervous system (59).

### 4.4 Immune and inflammatory responses

Regarding immune and inflammatory responses, we identified 8 proteins exhibiting decreased expression and 11 proteins exhibiting increased expression. The RNA helicase DDX3X belongs to the DEAD-box family. This protein is responsible for mRNA metabolism, which includes transcription, splicing, nuclear export, and translation (60). The role of this protein in the immune signaling pathway can be divided into three steps: (1) DDX3X is stimulated by pathogen-associated molecular patterns and interacts with NLRP3 to activate NLRP3 inflammasomes, leading to interleukin (IL)-1β production and activation of the pyroptosis pathway; (2) viral RNA stimulates DDX3X RNA helicase for assembly stress granules, leading to repression of cell survival; and (3) DDX3X plays roles similar to RIG-I–like receptors as a viral RNA sensor, induced through mitochondrial antiviral-signaling protein (MAVS), leading to type I interferon (IFN) production (61–64). Although several previous studies have suggested that DDX3X contributes to antiviral activity, in our study, its expression was down-regulated. Several other studies have found that viral proteins are involved in inhibiting the activity of DDX3X, which is one of the ways viruses adapt (65–67). Flightless I (FLII) is an abundant actin-binding protein from the gelsolin family that is distinguished by its ability to inhibit actin polymerization without severing actin filaments. This distinguishing trait places FLII at the core of the orchestration of several cellular processes necessary for physiological activities, such as wound repair, cancer growth, and inflammation. Its regulatory influence extends beyond cytoskeletal dynamics and to critical roles in cellular proliferation, differentiation, apoptosis, and migration. In addition, FLII finely regulates an assortment of cellular signaling pathways by acting both synergistically and competitively. Its association with two important inflammatory pathways, the NLRP3 inflammasome and the MyD88–Toll-like receptor (TLR)4 pathways, is especially important (68).

GCNT1 is a glycosyltransferase essential for *O*-glycan production. *O*-Glycans are complex carbohydrate molecules that bind to proteins via *O*-linked glycosidic bonds (69). A deficiency of GCNT1 was found to significantly impact E- and P-selectin–mediated inflammation involving myeloid cells while showing limited effects on the lymphoid system or adaptive immune responses (70).

EphA2 is a member of a family of receptor tyrosine kinases. Ivanov and Romanovsky (71) reported that expression of the EphA2 receptor and ephrin-B2 is increased in endothelial and epithelial cells in the initial phases of inflammation, suggesting they actively participate in processes such as the induction of adhesion molecule expression on cell surfaces and rearrangement of the intracellular cytoskeleton.

The role of NOS3 in the production of nitric oxide (NO) has emerged as a pivotal factor in host defense against viral infections. Up-regulation of this protein, along with NO synthases, has proved to be an effective mechanism for hindering virus multiplication. It is crucial to recognize that the impact of NO on host-virus interactions is multifaceted, affected by both time and concentration, as well as being contingent on the pathogenesis of the specific virus (72). Under certain circumstances, endogenous NO production may be inhibited, necessitating the introduction of exogenous NO to exert antiviral effects. Notably, maintaining the intricate balance between NO levels plays a significant role in modulating the immune response during viral infections. For instance, overproduction of NO promoted by inducible nitric oxide synthase downregulates the expression of inflammatory cytokines and chemokines. This downregulation is achieved through inhibition of both NF-κB and interferon regulatory factor (IRF)-1–dependent transcription in epithelial cells (73). Interaction between E3 ubiquitin and protein ligase RBBP6 reportedly negatively regulates Ebola virus replication via binding of RBBP6 to viral nucleoproteins. In addition, RBBP6 knockdown was shown to increase viral transcription and increase Ebola virus replication, whereas overexpression of RBBP6 or the peptide significantly inhibited both (74).

Certain zinc finger proteins, particularly those of the CCCH-type, constitute a diverse family that play critical roles in RNA metabolism, inflammation, and immunity (75). These proteins exhibit multifaceted functions, participating in various aspects of RNA processing and stability. Moreover, a subset of CCCH-type zinc finger proteins, including zinc finger antiviral protein, are significantly involved in antiviral defenses (76, 77). However, two of the proteins identified in our study, ZC3H4 and ZC3H6, were down-regulated, but antiviral or immune-suppressive properties of these proteins have not been reported. We highly expect that the discovery of these two proteins in our profile will be validated in relation to ZIKV infection in the future.

The mRNA processing body assembly factor LSM14A protein functions as an RNA processing body, binding to synthetic or viral RNA and DNA and mediating IRF3 activation and IFN induction for antiviral activity. Li et al. (78) reported that LSm14A is a sensor for both viral RNA and DNA and that it plays a critical role in initiating IFN production during the early stages of viral infection. In our study, up-regulation of this protein was indicative of up-regulation of the immune response.

LRRFIP1 binds to exogenous nucleic acids, amplifying the expression of IFN-*β*, and its expression is induced by both double-stranded RNA and double-stranded DNA. Importantly, the interaction of LRRFIP1 with β-catenin has emerged as a key mechanism that promotes the activation of β-catenin. This activation, in turn, enhances IFN-β expression by facilitating the binding of β-catenin to the C-terminal domain of the transcription factor IRF3, leading to increased production of type I IFN (79). In the present study, we identified MyD88, which serves as the central adaptor in inflammatory signaling pathways following activation of the TLR and IL-1 receptor families (80). Our study identified LRRFIP1 and MyD88 proteins as up-regulated, meaning that ZIKV-infected macaques overexpress immune defense pathway components.

CD36 plays a crucial role in the uptake of fatty acids and induces CD36 overexpression in macrophages and endothelial cells. This heightened CD36 expression is associated with increased intake of free fatty acids or oxidized low-density lipoprotein, contributing to alterations in lipid metabolism (81). Zhang et al. (82) reported that orexin A has a strong inhibitory effect on endothelial inflammation, especially when production of oxidized low-density lipoprotein is stimulated. Orexin A inhibits the attachment of monocytes (THP-1 cells) to endothelial cells, an important step in the inflammatory process. This effect is linked to the down-regulation of important vascular molecules such as vascular cell adhesion molecule-1, intercellular adhesion molecule-1, and E-selectin. Orexin A exerts its protective effects at the molecular level by targeting the MAP kinase p38 and NF-κB signaling pathways via its receptor, OX1R. The peptide efficiently inhibits MAP kinase p38 activation and prevents the phosphorylation of the important NF-κB cascade kinases IKK and IB. Importantly, orexin A inhibits translocation of p65 protein into the nucleus, emphasizing its involvement in inhibiting NF-κB–mediated inflammatory responses.

The expression of FLT4 and VEGFC is increased in macrophages after infection, and FLT4 signaling was found to be important in affecting key cellular processes, such as autophagy, inflammasome activation, and pyroptosis, all of which contribute to successful bacterial clearance (83).

Hypoxia-inducible factor (HIF) is an essential transcription factor that plays diverse roles in the complex landscape of immune responses. The significance of HIF in immunity is not confined to hypoxic environments, however; it also plays an important role during immunological responses and inflammation under normoxic conditions. Beyond its metabolic regulatory roles, HIF stabilization during normoxia directly regulates immunity-related gene expression, emphasizing its importance in immune cell function (84).

PI4KA plays an essential role in cellular physiology, particularly in the synthesis of phosphatidylinositol 4-phosphate. The resulting complex regulates the amounts of phosphatidylinositol 4-phosphate, a membrane phospholipid required for a variety of cellular activities (85). In addition, Tai and Salloum (86) reported that the discovery of PI4KA as an essential factor in HCV replication opened new possibilities for investigations into the precise interactions and signaling pathways involved in the formation of the membranous web.

The findings of Fletcher and Evans (87) demonstrated that MUC5B is not only required for the acute control of particle elimination in mucociliary clearance but also for the maintenance of homeostatic microbial composition and innate immune responses in the lungs. MUC5B deficiency induces an increase in culturable bacteria, as well as a shift in microbial diversity, most notably the acquisition of *Staphylococcus aureus*.

Cul3 is expressed in HIV-1 target cells, such as CD4+ T cells, monocytes, and macrophages. Overexpression of Cul3, on the other hand, results in a considerable reduction in HIV-1 infection, demonstrating its potential as a negative regulator of viral replication (88).

ARHGAP26 is a RHOA- and CDC42 GTPase–activating protein. RhoA and Cdc42 are Rho family small GTPases that regulate actin cytoskeletal construction, cell adhesion, migration, proliferation, and survival. Guo (89) investigated the effects of T cell–specific knockout of RhoA and Cdc42 on T cell development in the thymus, peripheral T cell homeostasis, activation, and differentiation to effector and regulatory T cells, in addition to T cell–mediated allergic airway inflammation and colitis, which play an important role in the pathogenesis of most, if not all, inflammatory diseases (89).

Mittelstadt et al. (90) reported that hDUS2 is a dsRNA-activated protein kinase regulator. Initially identified as a protein that interacts with PACT, hDUS2 also interacts with PKR via its dsRNA binding/dimerization domain, acting as a PKR kinase inhibitor. Activation of PKR suppresses protein synthesis globally through phosphorylation of eIF2 on serine. Prolonged PKR activation can lead to apoptosis.

We also identified OBSCN and ETNK2 as overexpressed. OBSCN plays a role in myofibrillogenesis, and ETNK2 is highly specific for ethanolamine phosphorylation. Two metalloendopeptidases we identified, ADAMTS5 and ADAMTSL1, were found to be down-regulated. Metalloendopeptidases play a variety of roles in tissue morphogenesis and pathological remodeling, inflammation, and vascular biology.

## 5 Conclusion

We conducted a serum proteomic analysis of wild stump-tailed macaques exhibiting the presence of ZIKV antibodies. We identified 52 serum proteins that exhibited significant differences in expression between seropositive and naïve groups. Among these proteins, five were implicated in anti-apoptosis mechanisms, leading to a reduction in apoptosis signaling pathway activity. Additionally, 11 proteins involved in viral activities and host defense mechanisms showed reduced expression levels, which would impact protein synthesis, DNA repair, and replication. Moreover, 12 proteins were found to be associated with neurological damage caused by ZIKV infection. Furthermore, 19 proteins were shown to be linked to immune responses and inflammation, whereas the remaining 5 proteins were associated with various other functions. These findings shed light on the intricate molecular responses of wild stump-tailed macaques to ZIKV infection and provide valuable insights for future research into potential therapeutic targets and diagnostic markers.

## Data Availability

The authors acknowledge that the data presented in this study must be deposited and made publicly available in an acceptable repository, prior to publication. Frontiers cannot accept a manuscript that does not adhere to our open data policies.

## Footnotes

^1^ https://bioinformatics.niaid.nih.gov/plaquereduction

^2^ https://www.hsls.pitt.edu/

^3^ https://www.genome.jp/kegg/ko.html

^4^ https://www.genome.jp/kegg/pathway.html

^5^ https://blast.ncbi.nlm.nih.gov/Blast.cgi

^6^ http://pantherdb.org/

^7^ http://stitch.embl.de/cgi/

^8^ https://string-db.org/
